# Associations Between Early Childcare Environment and Different Aspects of Adulthood Sociability: The 32-Year Prospective Young Finns Study

**DOI:** 10.3389/fpsyg.2019.02060

**Published:** 2019-09-10

**Authors:** Elli Oksman, Tom Rosenström, Kia Gluschkoff, Aino Saarinen, Mirka Hintsanen, Laura Pulkki-Råback, Jorma Viikari, Olli Tuomas Raitakari, Liisa Keltikangas-Järvinen

**Affiliations:** ^1^Department of Psychology and Logopedics, Faculty of Medicine, University of Helsinki, Helsinki, Finland; ^2^Unit of Psychology, Faculty of Education, University of Oulu, Oulu, Finland; ^3^Department of Clinical Physiology, Turku University Hospital, Turku, Finland; ^4^Department of Clinical Physiology and Nuclear Medicine, Turku University Hospital, Turku, Finland; ^5^Research Center of Applied and Preventive Cardiovascular Medicine, Faculty of Medicine, University of Turku, Turku, Finland; ^6^Faculty of Medicine, University of Turku, Turku, Finland; ^7^Research Centre of Applied and Preventive Cardiovascular Medicine, University of Turku, Turku, Finland

**Keywords:** sociability, early childcare environment, longitudinal study, personality development, personality assessment, multilevel modeling

## Abstract

Sociability is a widely studied trait that has been linked both with individual well- and ill-being. Although early childcare has been shown to affect social competence in children, its role in the development of different aspects of adulthood sociability is poorly understood. Using a longitudinal population-based sample (*N* = 464), this study investigated whether childcare arrangements at ages 3 or 6 are associated with self-reported adulthood sociability at ages 20 to 35 years. A total of five aspects of sociability were measured using three well-established personality inventories (EAS, NEO-FFI, and TCI). Multilevel modeling was applied to examine the association between early care and adulthood sociability, adjusting for several sources of random variation (between-individual variance, within-individual variance between measurement times, variance between used sociability indicators, and error variance that cannot be attributed to the previously mentioned) and potential confounders (disruptive behavior in childhood, parental socio-economic status, parent–child relationship quality, maternal age, and the number of children in the family). Based on our results, in comparison to home care, family daycare and center-based daycare at age 3 and center-based daycare at age 6 were associated with higher sociability later in life. The association was strongest for aspects of sociability that emphasize the willingness to be surrounded by other people and to be attached to them. In other words, characteristics of early care may contribute uniquely to the development of these aspects of sociability with effects that persist into adult life.

## Introduction

Sociability is a widely studied trait that can be found in all personality theories and inventories, in one form or another. High sociability (i.e., preference for a company instead of solitude) is typically associated with favorable outcomes and low sociability with harmful outcomes (Malouff et al., 2005; Hintsanen et al., 2009, 2011; Elovainio et al., 2015). Our understanding of the development of sociability traits and their early life etiologic factors remains, however, limited. Although prior work has established associations between early child care and the development of personality and social competence (Howes, 1988; Gluschkoff et al., 2018), there is a lack of longitudinal studies on the possible effects of child care arrangements on sociability in adulthood. As the majority of children in industrialized countries are cared for outside the home at least for some period of time, childcare arrangements offer a natural setting to study the role of different early care environments in the development of sociability. The unique characteristics of different care arrangements, such as opportunities to interact with peers or exposure to pedagogically planned early education, might play a role in the development of sociability. This study investigates early childcare as an exposure that could be linked to adulthood individual differences in overall sociability and its finer aspects.

### Sociability

Using a standard definition in the field, sociability generally refers to a preference for others’ company instead of solitude (Cheek and Buss, 1981) that is found to be adaptive for many social species (Silk, 2007; Dunbar, 2010; Silk et al., 2010), including humans. In humans, even newborn babies have an inborn need to seek others’ company and to bond with them (Ainsworth, 1989). Depending on the theoretical framework surrounding sociability, willingness to form relationships with others express a different kind of social need (Weiss, 1969). For example, some schools of sociology view close social relationships as important because through them the society organizes the individual’s thinking and acting (e.g., moral values, goals, even the sense of self). The more psychological view recognizes that people have several needs that only social relationships can satisfy, such as the need for recognition, for attention, for care, for belonging, for intimacy, and many more (Weiss, 1969). In other words, these social needs may partly explain the motivation for socially active behavior or higher sociability. The clearest form of sociability, a tendency to approach instead of withdrawing from others, can be measured rather easily by observation. In practice, however, people may have equal satisfaction with a few intense relationships or with a large number of relationships of lesser intensity – depending on social needs. The more complex constructs of human sociability, with elements related to both quantity and quality of social relationships (Plomin, 1976), are most often measured by self-evaluations due to their practicality and because they reflect the person’s self-concept (Robins et al., 2007).

The theoretical frameworks behind personality inventories differ – while some are developed by a consideration of the underlying biological and social determinants of individual differences (e.g., the biopsychological model by Cloninger et al., 1993), others are derived from a careful analysis of the personality assessment literature (e.g., the so-called Five Factor Model of personality; Costa and McCrae, 1992), yet, they all include a dimension for sociability. In some inventories, the trait has a different name (e.g., Reward Dependence or Extraversion), but its core content corresponds to the standard definition of sociability.

In most personality theories, sociability consists of correlated subcomponents related to different kinds of social needs. In other words, depending on the theoretic framework, the subcomponents emphasize different reasons *why* an individual prefers others’ company instead of solitude. For example, some subcomponents may focus more on dependency on others’ approval or tendency to feel emotionally attached to them (i.e., the quality of social relationships), whereas some emphasize the willingness to be surrounded by other people or the need to actively seek for as wide a social network as possible (i.e., the quantity of social relationships). These finer aspects usually strongly correlate with each other (Oksman et al., 2018), but may have differed developmental paths and associate with different outcomes.

As with any other personality trait, both genetic and environmental factors are implicated in the early development of sociability. Heritability studies estimate that approximately 20 to 65% of the within-population variation in sociability is of genetic origin (Buss and Plomin, 1986; Plomin et al., 1988; Cloninger et al., 1993). Thus far, attempts at understanding genetic etiology of sociability have not resulted in significant breakthroughs and previous genetic findings may be confounded and intertwined with environmental influences.

As a whole, social circumstances in childhood, including the home and care environment, have been associated with a variety of adulthood outcomes from environmental and socioeconomic status (SES) to psychological and physiological functioning (Power et al., 2002; Mäkinen et al., 2006). As for the environmental factors potentially associated with sociability, characteristics of the early environment have been shown to modify children’s later personality and their social competence (Nakao et al., 2000; Josefsson et al., 2013). Previous research has also established associations between early childcare and the development of personality traits, including prosociality (Brownell and Drummond, 2018) and dispositional compassion (Gluschkoff et al., 2018). In the current study, we examine if early childcare environments are also associated with adulthood sociability and its specific aspects. Investigating such early environmental factors may help shed light on the developmental mechanisms behind the different aspects of sociability.

### Early Childcare Environments

Early care environments can be roughly divided into those which take place at home or outside the home. In home care, the child is cared for at home either by a parent, relative, or a nanny, together with possible siblings. Home care is the most familiar care environment for a child and where most of the children are typically cared for at least for the first months of their life. However, home care can be challenging to arrange for parents after their financially supported parental leave has ended. Thus, the majority of children in industrialized countries are cared for outside of the home at least for some period (Waldfogel, 2001; Huston et al., 2015).

The most common forms of outside-home care are family daycare care and center-based daycare. In family daycare, the child is cared for at a caretaker’s home, usually with a small group of peers and in some cases, including the caretaker’s own children. Center-based daycare, in contrast, is pedagogically planned, goal-oriented early education with trained kindergarten teachers and with well-defined guidelines regarding, for example, peer group size and adult-to-child ratio.

Home care, family daycare, and center-based daycare can be distinguished by peer-group size, the level of caregiver training and the presence of pedagogical curriculum that includes developmental aims for the children, as well as by the presence of close attachment figures. Center-based daycare is the most strictly regulated early childcare environment with the most exposure to peers, whereas home care presents the most familiar, the least formal and the most individual form of care. Family daycare is something between the other two forms of care: the children are exposed to group-based care, but the group-size is usually smaller than in center-based daycare. Furthermore, in the 1980s, there were no official educational requirements for family daycare providers in Finland, even though some degree of training was strongly recommended. We acknowledge that these three early childcare environments do not, by all means, cover all the forms of childcare, but they present a rough and widely used distinction between the most common forms of care arrangements.

Previous research has shown that children’s early experiences in outside-home care can promote the development of both disadvantageous and favorable developmental outcomes, depending on the cumulative amount, the quality, and the timing of the childcare exposure (Maccoby and Lewis, 2003; Belsky, 2006; Vandell et al., 2010; Huston et al., 2015; Brownell and Drummond, 2018). Notably, conclusions from the previous evidence on early childhood education and care tend to vary between children who are under and over 3 years during the care environment exposure. For children aged 0–3 years the research evidence is more mixed, with some studies indicating benefits for outside-home care, some negative effects, and some studies reporting no effects at all (Melhuish, 2015). For example, several studies have shown that a high level of cumulative time spent in outside-home childcare, especially before the age of 4.5 years, is related to elevated levels of aggression, assertiveness, and disobedience in adolescence (Belsky, 2006; Vandell et al., 2010; Huston et al., 2015). By contrast, some studies have found that center-based daycare already in the infant and toddler years may contribute to the development of social competence and prosocial behavior (Brownell and Drummond, 2018), or lower levels of later emotional or behavioral difficulties compared to informal childcare (Gomajee et al., 2017). For children from 3 years upwards, more consistent results about the benefits of center-based daycare, preschool and other forms of group-based care have been explored in numerous studies (Melhuish, 2015). For example, a recent study found that at age 6 (but not yet at age 3), center-based daycare may increase dispositional compassion in adulthood (Gluschkoff et al., 2018).

Overall, previous findings suggest that the effects of early childcare depend both on the children’s developmental preparedness for outside-home care (e.g., metacognition and ‘theory of mind’ in the early years; Wellman et al., 2001; Chatzipanteli et al., 2014) and on the characteristics of the care environment, such as quality of care. Potential explanations for these findings involve the developmental unpreparedness of 0–3-year-old children to cope with social demands in care environments that are characterized by large peer groups. Still, at age 3, children typically have not developed sufficient knowledge or skills to engage in social interaction without constant adult guidance (Rubin and Pepler, 1995; Huston et al., 2015). Good quality and other structural features in an early care environment (e.g., group size and/or child-to-staff ratio, and training, permanence, sensitivity and responsivity of caretakers) have been raised as one major explanation for findings where center-based daycare may be beneficial even when the child is less than 3 years old (Brownell and Drummond, 2018). However, the ‘good quality’ of center-based childcare can be challenging to achieve and maintain in practice. That, in turn, places more pressure on the child’s developmental preparedness to cope in the care environment. After the age of 3 to 4 years, playing tends to be more interactive and less dependent on guidance (Hughes, 2011), and interacting with peers has a more central role in a child’s development (Harris, 1995). Thus, especially when the child has developed a sufficient level of cognitive preparedness, center-based care is more likely to be beneficial for a child’s later social development and may promote children’s positive behavior in early social interactions (Huston et al., 2015; Melhuish, 2015).

Although outside-home care has become the norm in many modern societies and despite the vast interest in how early childcare shapes a child’s development, findings on the longitudinal effects of different childcare environments are still limited. Namely, most of them have a follow-up period that covers only adolescence. As the largest changes in personality tend to occur in young adulthood (ages 20–40; Roberts et al., 2006), studies spanning over this age period would increase knowledge whether early environmental effects can be detectable despite these normative changes. Furthermore, studies of this kind often focus on early antisocial (criminal) tendencies or (lack of) social competence rather than on normative personality development. In the current paper, we have two aims: (1) to investigate the extent to which different childcare environments at age 3 or 6 are associated with self-evaluated sociability at ages 20 to 35 years, and (2) with what kind of aspects of sociability are these associations found. Early childcare is a natural setting in which to study not only the effects of exposure to different types of care environments but also the effects of the timing of given childcare exposure on a child’s personality development later.

## Materials and Methods

### Participants

The participants were derived from the ongoing population-based Cardiovascular Risk in Young Finns Study, or Young Finns Study for short, which is one of the largest follow-up studies on cardiovascular risk from childhood to adulthood (Raitakari et al., 2008). The main aim of the Young Finns Study is to determine the contributions made in childhood lifestyle, biological, and psychological measures to the risk of cardiovascular diseases in adulthood. In addition to cardiovascular health, the data enables the study of personality in the participants exceptionally widely, both longitudinally and using several different personality assessments. The original sample (*N* = 3,596) were healthy children and adolescents randomly selected from six age-based cohorts (ages 3–18 years at the baseline in 1980) which have now been followed for 32 years. The study was approved by the local ethics committee, the Finnish National Advisory Board on Research Integrity (TENK) appointed by the Ministry of Education and Culture, and it was conducted in accordance with the Helsinki declaration. Informed written consent was provided from the parents when participants were still under-aged (i.e., for measurement waves 1980 and 1983 in the present study) and after reaching adulthood, from the participants themselves (i.e., in 1997–2012).

The participants of the present study consist of the youngest age cohort, born in 1977 (*n* = 577). Only this age cohort had information about their childcare arrangements both in 1980 and 1983 when the participants were, on average, 3 and 6 years old. In practice, participant age ranged from 2 years 9 months to 3 years 11 months in the first wave and from 5 years 9 months to 6 years 11 months in the second wave. First, we excluded participants who did not exclusively attend home care, family daycare, or center-based care (the most common forms of care), and those with a non-specified form of care (e.g., *“[The child is] at home without a caregiver*” or “*Other daycare arrangement [not specified],” n* = 174 in total). A total of 360 participants (62%) had full information for all the study variables and covariates derived from childhood (in 1980 and 1983), and at least one measurement for each aspect of sociability measured in 1997–2012. Males were more likely to have missing observations; otherwise, data were missing at random. All the analyses were performed both with and without missing data modeling (Vandell et al., 2010) and the results were similar using both methods. All descriptive statistics and analyses reported are based on estimates employing missing data modeling to minimize the possible effects caused by missing information.

### Measures

#### Early Childcare Environment

In the present study, we decided to focus on the three most common forms of care: home care, family daycare, and center-based daycare (Table 1). We did this by creating a categorical variable consisting of three non-overlapping forms of care based on the childcare arrangements reported by a parent (“*How is child care arranged?*”) in 1980 (at age 3) and 1983 (at age 6). In home care, the participants had been cared for at home by a parent, a relative, or a nanny. Family daycare refers to a caring environment where the participants had been cared for in another family, typically at a caretaker’s home, and where they were accompanied by two to four other children, not including the caretaker’s own children. In the 1980s, there were no official educational requirements for family daycare providers, although some degree of training was strongly recommended. Center-based daycare as a care environment, by contrast, is characterized by more strictly regulated standards regarding, for example, caregivers per child ratio, educational level of kindergarten teachers, pedagogical goals, and the size of peer-groups (per guidelines, a maximum of 12 children per group with 3-year-olds and 16 with older children).

**TABLE 1:** Descriptive statistics of the study variables (*N* = 464).

	***M* (*SD*)**		**Data collection intervals (*M* [*SD*] or *N* [%])**					
			
**Measure (range)**	**or%**	***N*_*y[i]*_**	**1980**	**1983**	**1997**	**2001**	**2007**	**2012**
Gender (0 = women, 1 = men)	52%	464	–	–	–	–	–	–
**Early childcare environment**								
Home care	–	–	264 (57%)	234 (50%)	–	–	–	–
Family daycare	–	–	118 (25%)	65 (14%)	–	–	–	–
Center-based daycare	–	–	82 (18%)	165 (36%)	–	–	–	–
**Adulthood sociability (1**–**5)**								
EAS: Sociability	3.38 (0.85)	1856	–	–	3.53 (0.81)	3.46 (0.82)	3.27 (0.85)	3.26 (0.87)
NEO-FFI Extraversion	3.37 (0.62)	928	–	–	–	–	3.38 (0.63)	3.36 (0.62)
TCI RD1: Sentimentality	3.05 (0.67)	1856	–	–	3.11 (0.64)	3.12 (0.63)	3.00 (0.69)	2.96 (0.70)
TCI RD3: Social attachment	3.59 (0.86)	1856	–	–	3.63 (0.83)	3.66 (0.84)	3.56 (0.86)	3.50 (0.89)
TCI RD4: Dependence	3.30 (0.62)	1856	–	–	3.18 (0.60)	3.33 (0.61)	3.34 (0.60)	3.35 (0.65)

*EAS, Emotionality-Activity-Sociability Temperament Survey; NEO-FFI, The Neuroticism-Extraversion-Openness Five-Factor Inventory; TCI, Temperament and Character Inventory. At the baseline in 1980, the participants (born in 1977) were 3-year-olds and during the latest follow-up in 2012, 35 years old. Home care: care at home by a parent, a relative, or a nanny. Family care: care at the care provider’s home with a maximum of four children. Center-based care: care in kindergarten with a maximum group size of 12 (at age 3) and 16 (at age 6). All the values presented are based on estimates employing missing data modeling.*

Typically, children in Finland attend preschool at age 6 and the official age for school entry is 7 years. However, in the 1980s, preschools were still only partially introduced in Finland, and those 6-year-olds who were in outside-home care typically attended either family care or center-based care. For some of our participants, center-based daycare may have corresponded to pre-school, but because “preschool” was not a specified care arrangement option in the questionnaire that was used in the Young Finns Study in 1980 and 1983, it is not possible to separate these participants from those who still attended center-based daycare. In practice, these two forms of care are close to each other both physically and in content: pre-schools are typically situated in the same or nearby building as center-based daycare, and the children who attended pre-school in the morning typically changed to center-based daycare during the afternoon.

#### Adulthood Sociability

In the Young Finns Study, adulthood personality has been self-evaluated by three different personality inventories which provides an extraordinary opportunity to compare them using the same individuals. A total of five sociability indicators were derived from these three commonly used personality inventories: Sociability, Extraversion, Sentimentality, Social attachment, and Dependence. Sociability was measured with five items (e.g., ‘*I like to be with people’*; Cronbach’s alpha varied between α = 0.77–0.82 over the measurement occasions) using Buss and Plomin’s Emotionality-Activity-Sociability Temperament Survey (EAS; Buss and Plomin, 1975, 1986). The scale assesses a tendency to prefer and enjoy the presence of others over being alone, and how comfortable a person feels in a group. Extraversion was measured with 12 items (‘*I really like to discuss with people’*; α = 0.81–0.82) using Neuroticism-Extraversion-Openness Five-Factor Inventory (NEO-FFI; McCrae and Costa, 1988; Costa and McCrae, 1992). The trait refers to warmth, gregariousness, assertiveness, activity, excitement seeking, and positive emotions. Sentimentality (10 items, ‘*I like to please other people as much as I can*’; α = 0.70–0.76), Social Attachment (eight items, ‘*I would like to have warm and close friends with me most of the time’*; α = 0.82–86), and Dependence (six items, ‘*I don’t care very much whether other people like me or the way I do things* (reverse scored)’; α = 0.46–0.63) were measured using subscales of the Reward Dependence scale in Temperament and Character Inventory (TCI; Cloninger, 1987; Cloninger et al., 1993). Sentimentality refers to a tendency to be deeply moved by emotional appeals and to an inclination to show, share, and adapt emotions easily in the presence of others; Social Attachment to a person’s tendency to prefer company and intimacy over solitude and privacy; and Dependence to a person’s need for emotional support and approval from others, combined with a tendency to please and be preoccupied with fears of being abandoned. These five aspects do not cover all the variability in sociability but present an example of both the quality and quantitative side of sociability and demonstrate similarities and differences between three commonly used personality inventories in regard to sociability.

In the Young Finns Study, NEO-FFI scales have been measured twice (Table 1; age range of 30–35 years), and TCI and EAS scale four times (age range of 20–35 years). With all these inventories, a five-point precision ranging from 1 (*Definitely false*) to 5 (*Definitely true*) was used from 1997 to 2001. From 2007 onwards, the response options were slightly modified to have a range from 1 [(*The definition fits me) poorly or not at all*] to 5 *[(The definition fits me) very well]*. A mean score for each aspect of the indicator was calculated for those participants who did not have more than one missing item. All indicators of adulthood sociability correlated with each other (*r* = 0.10–0.59, *p* < 0.003). Responses to all the sociability indicators were combined to represent ‘overall adulthood sociability’ (see section “Statistical Analyses”). After analyzing overall adulthood sociability, the five indicators of sociability were analyzed separately because they represent different aspects of sociability.

#### Covariates

All the constructed multi-level models (see below) were adjusted for nine covariates in total: for gender, for childhood home environment (parental SES, maternal age, the number of children in the family), for disruptive behavior in childhood (aggression, hyperactivity, lack of social adjustment), and parent–child relationship quality (emotional warmth and acceptance toward the child). Adjustment of these factors removes effects of some potential causes for non-random selection to daycare groups, thereby helping causal interpretations based on this naturally occurring experiment. All possible confounding factors cannot be removed, but these nine present covariates that have previously been widely studied in association with later personality development and social behavior (e.g., Nakao et al., 2000; Josefsson et al., 2013; Safra et al., 2016; Laible et al., 2017; Dobewall et al., 2018; Gluschkoff et al., 2018).

For all the covariates, the scores from 1980 and 1983 were averaged. SES was measured by the total family income, and by parents’ years of education and parental occupational status. These variables were standardized, summed, and standardized again in order to form the variable for parental SES, thereby giving equal weight to income and education variation (see Text-supplement S1 in Rosenström et al., 2012, for further details). Participant’s disruptive behavior in childhood, a scale derived from the Health Examination Survey (Wells, 1980), presents a form of the child’s unpreparedness and challenges faced in being in peer-groups and group-based care environment. The scale contains three domains: aggression, hyperactivity, and lack of social adjustment. A child’s aggression (six items) includes the aggressive behavior perceived by peers (e.g., “Other children frequently accuse him or her of fighting”), by other adults (e.g., “Parents of other children have complained about his or her behavior”), and by the child’s own parents (e.g., “‘Accidentally’ hits, trips or shoves other children”). In 1980 the aggression item’s scale was yes/no; in 1983 a five-point Likert-scale was used, from 1 (*totally disagree*) to 5 (*totally agree*). For better comparability, both were scaled to have a range of 0 to 1 where a higher value indicates greater aggression (α = 0.62). A child’s activity was evaluated on a four-point scale ranging from “1: *He or she stays calm even after most other children have become restless*” to “4: *He or she is always on the move, talks non-stop, and his or her activity is striking*.” A child’s lack of social adjustment was reported with one item on a three-point scale (“1: *He or she survives well in everyday life*; 2: *His or her behavior does not worry you*; 3: *Occasionally, his or her behavior worries you. You think of him or her as a problem child or are afraid that he or she may become one*”). The parent–child relationship quality scale was developed based on the Operation Family Study (Makkonen et al., 1981). It contains two child-rearing components: emotional warmth and acceptance toward the child. Emotional warmth was measured using four items [e.g., “The child is significant to me,” from 1 (*not significant*) to 5 (*very significant)*; α = 0.74], and acceptance toward the child was measured with three items [e.g., “In difficult situations, the child is a burden,” from 1 (*totally disagree*) to 5 (*totally agree*); α = 0.71].

### Statistical Analyses

We used multilevel regression modeling as the method that recognizes the hierarchical structure of the data, such as dependencies between repeated outcome measurements in adulthood (Gelman and Hill, 2007; Dingemanse and Dochtermann, 2012). Furthermore, this method reveals whether exposure to childcare is differentially associated with different dependency structures, or random-effects, of the data. We did this by partitioning the population variance in adulthood sociability to trait (between-individual) variance, differences among used inventories (sociability indicator variance), the time-variant part of the overlapping variance of inventories in overall sociability (within-individual variance), and measurement error or idiosyncratic differences that cannot be attributed to an individual, to follow-up or to a sociability indicator. The effect of the childcare environment was analyzed separately at the age of 3 and 6 years. All the analyses were based on pooled multiple imputation estimates using chained equations (van Buuren and Groothuis-Oudshoorn, 2013) and the variables were standardized before being entered into the model. Variance Inflation Factors indicated no multicollinearity problems in any of the models, with all the factors being less than 10 (Mitra, 2007).

First, we predicted the overall adulthood sociability by exposure to different childcare environments while simultaneously controlling for all the covariates. Second, we predicted each of the five sociability indicators separately. In regression models, home care was established as the reference group, corresponding to an intercept. We also tested if the results would hold even after adjusting for daycare history (i.e., care arrangement at age 3 and 6). This resulted in nine different combinations of daycare history. However, in some of the groups (namely, in those whose child were in outside-home care at age 3 and in in-home care at age 6) the number of observations was less than 5%, which easily leads to high variance estimates and low reliability of the results. Therefore, we concentrated on the main effects of the daycare environment (i.e., either at age 3 or at age 6). The results of daycare history, which should be regarded as indicative, are presented as Supplementary Material. All statistical analyses were done in R software version 3.3.2., supplemented with a MICE package, version 2.25 for imputation analysis (van Buuren and Groothuis-Oudshoorn, 2013) and a lme4 package version 1.1–12 for multilevel regression analyses (Bates et al., 2015).

## Results

### Overall Adulthood Sociability

The descriptive statistics of sociability indicators and the distribution of participants across different forms of care are presented in Table 1. Table 2 shows the association between early care arrangements and adulthood sociability. Family daycare and center-based care at age 3 were independently associated with overall adulthood sociability. Relative to home care, exposure to family daycare (β = 0.19, 95% CI 0.05 to 0.29, *p* = 0.007) or center-based daycare (β = 0.21, 95% CI 0.06 to 0.34, *p* = 0.014) at age 3 predicted a higher degree of overall adulthood sociability later in life. In contrast, at age 6, only those who were cared for in a center-based daycare had higher overall adulthood sociability compared to home care (β = 0.21, 95% CI 0.07 to 0.30, *p* = 0.004). When the childcare at age 6 was adjusted for child care status at age 3, daycare history of outside-home care (family daycare or center-based daycare) at age 3 combined with center-based daycare at age 6 associated with higher overall sociability in comparison to home care at age 3 and 6 (see Supplementary Table S1). Male gender was the only adjusting covariate that was independently associated with overall adulthood sociability. Men had on average 0.44 standard deviations lower overall adulthood sociability than women (*p* < 0.001).

**TABLE 2:** Multilevel regression analyses of early childcare environment at age 3 and 6 predicting mean levels of standardized sociability indicators and overall adulthood sociability.

	**Early childcare environment**	**Childcare environment at age 3**			**Childcare environment at age 6**		
			
**Sociability indicator**		**β**	**95% CI**	***p*-value**	**β**	**95% CI**	***p*-value**
EAS: Sociability	Home care	(Ref)			(Ref)		
	Family care	**0.32^∗∗^**	0.07 to 0.46	0.005	0.16	−0.01 to 0.46	0.280
	Center-based care	**0.29^∗^**	0.03 to 0.50	0.037	**0.28^∗^**	0.11 to 0.49	0.017
NEO-FFI	Home care	(Ref)			(Ref)		
Extraversion	Family care	0.17	−0.06 to 0.29	0.094	0.11	−0.08 to 0.32	0.406
	Center-based care	0.20	−0.00 to 0.41	0.108	0.20	−0.02 to 0.32	0.073
TCI: RD1	Home care	(Ref)			(Ref)		
Sentimentality	Family care	0.08	−0.04 to 0.22	0.372	0.07	−0.08 to 0.23	0.452
	Center-based care	0.14	−0.02 to 0.30	0.130	0.14	−0.04 to 0.22	0.084
TCI: RD3	Home care	(Ref)			(Ref)		
Social attachment	Family care	**0.33^∗∗^**	0.09 to 0.47	0.005	0.23	−0.07 to 0.39	0.130
	Center-based care	**0.37^∗^**	0.16 to 0.62	0.011	**0.31^∗^**	0.06 to 0.44	0.014
TCI: RD4	Home care	(Ref)			(Ref)		
Dependence	Family care	0.03	−0.05 to 0.21	0.705	0.06	−0.05 to 0.26	0.535
	Center-based care	0.05	−0.13 to 0.18	0.612	0.12	−0.02 to 0.23	0.133
Overall adulthood	Home care	(Ref)			(Ref)		
sociability	Family care	**0.19^∗∗^**	0.05 to 0.29	0.007	0.13	0.00 to 0.28	0.157
	Center-based care	**0.21^∗^**	0.06 to 0.34	0.014	**0.21^∗∗^**	0.07 to 0.30	0.004

*The statistically significant values are bolded, ^∗^ = *p* < 0.05, ^∗∗^ = *p* < 0.01. EAS, Emotionality-Activity-Sociability Temperament Survey; NEO-FFI, The Neuroticism-Extraversion-Openness Five-Factor Inventory; TCI, Temperament and Character Inventory. NEO-FFI was measured twice (2007, 2012; age range 30–35 years; *N_*y[i]*_* = 928), EAS and TCI four times (1997, 2001, 2007, 2012; age range 20–35 years; *N*_*y[i]*_ = 1,856 for each trait). Overall adulthood sociability consists of all the five sociability indicators (*N*_*y[i]*_ = 8,352 in total). The *p*-value indicates the difference from home care which was set as a reference group. Models were done separately for each adulthood outcome, and they all were adjusted for gender, disruptive behavior in childhood, parental socio-economic status, parent-child relationship quality, maternal age, and the number of children in the family. All the values presented are based on estimates employing missing data modeling.*

Table 3 presents the associations between early care and adulthood sociability separately for men and women. Namely, at age 3, family care increased overall adulthood sociability in men (β = 0.22, 95% CI −0.01 to 0.34, *p* = 0.034) and center-based care in women (β = 0.27, 95% CI 0.10 to 0.50, *p* = 0.021) in comparison to home care. At age 6, center-based care associated with higher adulthood sociability only in women (β = 0.24, 95% CI 0.09 to 0.41, *p* = 0.018). Men had a similar but weaker trend, possibly due to a smaller number of observations (*N_*y[i]*_* = 3,978 for men and 4,374 for women).

**TABLE 3:** Multilevel regression analyses of early childcare environment at age 3 and 6 predicting mean levels of standardized sociability indicators and overall adulthood sociability separately for men and women participants.

	**Early childcare environment**	**Men**			**Women**		
			
**Sociability indicator**		**β**	**95% CI**	***p*-value**	**β**	**95% CI**	***p*-value**
EAS: Sociability	**At age 3**						
	Home care	(Ref)			(Ref)		
	Family care	**0.41^∗^**	0.00 to 0.57	0.016	0.29	0.01 to 0.57	0.063
	Center-based care	0.19	−0.13 to 0.52	0.296	0.39	−0.01 to 0.67	0.052
	**At age 6**						
	Home care	(Ref)			(Ref)		
	Family care	0.15	−0.10 to 0.56	0.477	0.18	−0.07 to 0.60	0.382
	Center-based care	0.22	−0.09 to 0.47	0.202	**0.35^∗^**	0.14 to 0.69	0.037
NEO-FFI	**At age 3**						
Extraversion	Home care	(Ref)			(Ref)		
	Family care	0.21	−0.09 to 0.38	0.159	0.16	−0.12 to 0.38	0.302
	Center-based care	0.18	−0.11 to 0.44	0.282	0.20	−0.06 to 0.55	0.255
	**At age 6**						
	Home care	(Ref)			(Ref)		
	Family care	0.12	−0.09 to 0.46	0.477	0.10	−0.16 to 0.44	0.595
	Center-based care	0.19	−0.08 to 0.38	0.201	0.20	−0.05 to 0.44	0.242
TCI: RD1	**At age 3**						
Sentimentality	Home care	(Ref)			(Ref)		
	Family care	0.18	−0.10 to 0.31	0.135	–0.02	−0.10 to 0.24	0.869
	Center-based care	0.22	−0.11 to 0.37	0.110	0.06	−0.06 to 0.36	0.625
	**At age 6**						
	Home care	(Ref)			(Ref)		
	Family care	0.02	−0.28 to 0.20	0.873	0.08	−0.04 to 0.38	0.517
	Center-based care	0.19	−0.12 to 0.28	0.099	0.10	−0.07 to 0.27	0.345
TCI: RD3	**At age 3**						
Social attachment	Home care	(Ref)			(Ref)		
	Family care	0.27	−0.08 to 0.47	0.098	**0.41^∗^**	0.11 to 0.66	0.015
	Center-based care	0.15	−0.18 to 0.44	0.402	**0.58^∗∗^**	0.29 to 0.96	0.005
	**At age 6**						
	Home care	(Ref)			(Ref)		
	Family care	0.08	−0.21 to 0.42	0.692	0.37	−0.08 to 0.60	0.102
	Center-based care	0.23	−0.07 to 0.46	0.158	**0.40^∗^**	0.07 to 0.62	0.027
TCI: RD4	**At age 3**						
Dependence	Home care	(Ref)			(Ref)		
	Family care	0.02	−0.12 to 0.27	0.873	0.05	−0.09 to 0.27	0.659
	Center-based care	0.02	−0.26 to 0.18	0.868	0.10	−0.12 to 0.33	0.494
	**At age 6**						
	Home care	(Ref)			(Ref)		
	Family care	0.06	−0.08 to 0.36	0.660	0.08	−0.11 to 0.32	0.552
	Center-based care	0.10	−0.14 to 0.24	0.404	0.15	−0.01 to 0.34	0.162
Overall adulthood	**At age 3**						
sociability	Home care	(Ref)					
	Family care	**0.22^∗^**	−0.01 to 0.34	0.034	0.18	0.04 to 0.36	0.064
	Center-based care	0.15	−0.09 to 0.31	0.187	**0.27^∗^**	0.10 to 0.50	0.021
	**At age 6**						
	Home care	(Ref)			(Ref)		
	Family care	0.08	−0.08 to 0.32	0.515	0.17	−0.00 to 0.39	0.183
	Center-based care	0.18	−0.04 to 0.30	0.069	**0.24^∗^**	0.09 to 0.41	0.018

*The statistically significant values are bolded, ^∗^ = *p* < 0.05, ^∗∗^ = *p* < 0.01. NEO-FFI, The Neuroticism-Extraversion-Openness Five-Factor Inventory; TCI, Temperament and Character Inventory; EAS, Emotionality-Activity-Sociability Temperament Survey. NEO-FFI was measured twice (2007, 2012; age range 30–35 years; *N_*y[i]*_* = 442 for men and 486 for women), TCI and EAS four times (1997, 2001, 2007, 2012; age range 20–35 years; *N*_*y[i]*_ = 884 for men and 972 for women for each trait). Overall adulthood sociability consists of all the five sociability indicators (*N_*y[i]*_* = 3978 for men and 4374 for women in total). The *p*-value indicates the difference from home care which was set as a reference group. Models were done separately for each adulthood outcome, and they were all adjusted for disruptive behavior in childhood, parental socio-economic status, parent-child relationship quality, maternal age, and the number of children in the family. All the values presented are based on estimates employing missing data modeling.*

Regarding the random effects, the between-individual variance with 3-year-olds was 0.24 (95% CI 0.20 to 0.27), repeated-measurements (i.e., within-individual) variance in overall sociability was 0.01 (CI 0.00 to 0.03), the sociability indicator variance was 0.08 (CI 0.03 to 0.30), and residual/error variance that cannot be attributed to an individual, to follow-up, or to a sociability indicator was 0.62 (CI 0.60 to 0.64). In other words, between-individual differences, within-individual changes, temporally stable differences between the sociability indicators, and measurement errors accounted for 25, 3, 8, and 65% of the variance in overall adulthood sociability, respectively. The error variance includes both measurement errors in individual indicators as well as within-individual changes that are not consistent across the indicators (i.e., do not reflect overall sociability nor stable indicator-specific differences). These results were similar for the model with the care environment at age 6 as predictors.

### Different Aspects of Adulthood Sociability

When the five indicators of sociability were analyzed separately, family daycare and center-based care at age 3 predicted higher adulthood Sociability (derived from EAS) and Social Attachment (TCI) in comparison to those who have been cared for at home (Table 2). At age 6, only center-based daycare predicted higher Sociability and Social Attachment. Regarding the daycare history, outside-home care at age 3 together with center-based care at age 6 predicted higher adulthood outcome only with these two aspects of sociability (see Supplementary Table S2). A similar but statistically weaker trend was present for Extraversion (NEO-FFI) and Sentimentality (TCI; *p* = 0.073 and 0.084, respectively) both with the main effects and with the supplementary analysis of daycare history. Dependence (TCI) was not predicted by daycare. From covariates, male gender was associated with lower adulthood outcome with all the other sociability indicators except Extraversion. With Extraversion (NEO-FFI), the child’s lack of social adjustment both at age 3 and 6 (β = −0.11, 95% CI −0.16 to −0.01, *p* = 0.027 in both cases) was the only predictor that associated with the outcome.

In the analyses performed separately for men and women (Table 3), an early care environment at age 3 and 6 associated with higher adulthood Social attachment (TCI) only in the women’s subsample. Regarding Sociability (EAS), at age 3, family care predicted a higher adulthood outcome only in men whereas in women, both family care and center-based care had a positive, yet statistically weaker, trend on adulthood outcome.

## Discussion

The purpose of this study spanning over 32-years was to examine if the childcare environment at age 3 or age 6 is associated with self-reported adulthood sociability, and if these associations depended on the specific indicator of sociability. We focused on three forms of care that present the most common forms of care: home care, family daycare, center-based daycare. These three childcare arrangements can be intrinsically distinguished by peer-group size, the familiarity of the environment, the level of caregiver training and the presence of pedagogical curriculum that includes developmental aims for the children. Furthermore, The Young Finns Study sample gave us a special opportunity to investigate the association between these early daycare environments with several aspects of sociability derived from three commonly used personality inventories with the same participants. Whereas sociability as a whole is defined as a willingness to be with others instead of in solitude, the finer aspects of sociability provide more insights of the motivation for seeking others’ company, such as quality or quantity of social interactions. Our results showed that group-based outside-home care associated with higher overall adulthood sociability. With center-based daycare, we found this association both for 3- and 6-year-olds. The association was strongest for aspects of sociability that emphasize the willingness to be surrounded by other people and to be attached to them.

Previous studies have shown that exposure to center-based daycare may predict several developmental outcomes, both beneficial and harmful (Maccoby and Lewis, 2003; Belsky, 2006; Vandell et al., 2010; Huston et al., 2015). Most often, center-based care from age 0 to 3 or even to 4.5 years has been seen as a risk for child’s later social development (Belsky, 2006; Vandell et al., 2010). In contrast, some studies have shown that if center-based daycare is of sufficient quality, it may do no harm or even be beneficial for the socio-emotional development of children under the age of 3 (Gomajee et al., 2017; Brownell and Drummond, 2018). It might be that, due the limited self-control, theory of mind, or language capabilities of a child, the quality of childcare (e.g., peer group sizes and adult-to-child ratio) tends to matter more in the toddler- than in the preschool years, regardless of the form of care. That, in turn, may partly explain why previous findings with children aged 0–3 years vary more than with older children for which the evidence of the benefits of group-based outside-home care to the child’s later development is more coherent (Melhuish, 2015). In other words, children closer to the preschool age can cope in groups more independently than toddlers, and thus they are likely to be less dependent on the quality of care. However, none of these studies have focused on the possible effects of the childcare environment on the development of adulthood sociability.

Based on our results, 3-year-olds in both family daycare (consisting of a group of 2 to 4 children who are taken care of at the caretaker’s home, not including the caretaker’s own children) and center-based daycare (typically consisting of a group of 12 children) associated with higher adulthood sociability in comparison to home care. In other words, despite the difference in peer-group sizes, home care and center-based daycare did not differ in their association with sociability when participants were 3 years old. In general, Nordic countries have an early childcare system which is considered to be of high quality on an international level (Melhuish, 2015). In Finland, the municipal daycare is generally homogeneous and of high quality in regard to, for example, caregivers’ educational level, child-to-adult ratios, and other daycare conditions that are based on prevailing regulations that early childcare providers are bound to follow. Thus, our result with 3-year-olds are in line with previous findings where group-based care with sufficient quality may do no harm or, more interestingly, may even be beneficial for later development.

In contrast, 6-year-olds benefited relatively more only from the exposure to center-based daycare (typically consisting of a group of 16 children) in comparison to home care in regard to their later development of sociability. The results are logical not only because of better developmental preparedness of 6-year-olds compared to 3-year-olds but also because center-based daycare, which in the case of most of the 6-year-olds corresponds to preschool, is a caring environment which focuses on preparing children to the transition to school.

As previously discussed, sociability has a general definition as a preference for others’ company instead solitude, which is widely used in both an animal and human context. However, with humans, sociability is seen as a more complex construct and by using different methods besides observation, like self-evaluations, it is possible to separate finer aspects of human sociability. Namely, some aspects of sociability emphasize the quantity of social relationships and activity to seek others’ company (in this study, EAS Sociability and NEO-FFI Extraversion), whereas other aspects emphasize the quality of social relationships, such as dependence on others’ company and warm social attachment (such as TCI Reward Dependence’s subscales Sentimentality, Social attachment, and Dependence used in our study). Even though the aspects of sociability used in the present study cover only some examples of these differences and the diversity between them, they have been derived from some of the most widely used personality inventories.

Previously, the diversity of the same self-evaluated aspects of sociability used in the present study were acknowledged (Oksman et al., 2018). In the present study, variation between the sociability indicators was almost three times more than the within-individual change from age 20 to 35 (8 vs. 3%). Thus, in addition to examining overall sociability, it was justified to study the different indicators of sociability separately. With 3-year-olds, childcare arrangements that took place outside the home (i.e., family daycare and center-based daycare) associated especially with the kind of adulthood sociability that emphasizes preference to be surrounded by other people (i.e., EAS Sociability) and willingness to be socially attached to them (i.e., TCI RD3: Social Attachment). At the age of 6, only center-based daycare increased EAS Sociability and RD3: Social Attachment in adulthood in comparison to those who were cared for at home. A similar, though statistically non-significant, positive trend of center-based daycare at the age of 6 was also present for NEO-FFI Extraversion (i.e., preference to actively seek for others’ company) and TCI RD1: Sentimentality (i.e., tendency to share mental states and emotions with others). In other words, based on our study, the early care environment associated both with quantitative and qualitative elements of later sociability.

There can be several reasons why participants who, in childhood, were cared for outside the home are more sociable in adulthood than individuals who were cared for at home. More interestingly, there can be several reasons why any type of outside-home care at age 3 and only center-based daycare at age 6 associated with higher adulthood sociability. One possibility is that at age 3, exposure to any kind of group care environment encourages the development of later sociability (i.e., being in a group increases preference for others’ company), whereas at age 6, only center-based daycare (or, preschool) promotes the development of higher sociability. In addition to providing exposure to peer-groups, center-based daycare involves an early education and pedagogical environment that encourages and models behaviors that are probably relevant for the development of sociability.

Many other studies have previously acknowledged how women as a whole tend to have higher sociability than men (Feingold, 1994; Brändström et al., 2001; Costa et al., 2001; Miettunen et al., 2007; Lippa, 2010; Weisberg et al., 2011; Oksman et al., 2018). This was also noted in the present study. Furthermore, we noticed some gender-specific trends regarding the early care environment and different aspects of adulthood sociability. Namely, associations between outside-home care and adulthood TCI Social attachment was clearer in women, and for men, family care associated with adulthood outcome (overall adulthood and EAS Sociability) more often than center-based care. The results could be explained by different evolutionary and sociocultural roles that men and women have. From an evolutionary perspective, both animal and human research identifies that the peptide hormone oxytocin, which is strongly modulated by primary female sex hormone estrogen, is related to social bonding, attachment, and affiliation (Campbell, 2008). In other words, women on average have a better biological preparedness for this kind of social behavior which is typically needed in a nurturing context compared to men. However, nurturing-relevant social behavior represents only a small part of sociability, defined as a willingness to be with others instead of solitude. Maccoby’s (1990) theory of the development of gender-typed behaviors present more sociocultural perspectives to the gender differences. She argues that gender differences in individual characteristics, such as sociability, are minimal when children are observed individually. Rather, gender differences in social behavior develop from social interaction, particularly from same gender peer groups. In such groups, gender-specific interaction styles and roles start to emerge. Namely, higher sociability of women may emerge because women are expected to be more nurturing, tender-minded or more “warm” and orientated toward other people than men are (Costa et al., 2001; Lippa, 2010; Weisberg et al., 2011). Center-based daycare may offer more opportunities for children to find same-gender peer-groups than home or family care environments do, thus also exposing children to gender-specific expectations both from peers and caregivers. This, in turn, might encourage the development of gender-specific roles that boost the development of sociability especially in girls.

### Strengths and Limitations

The present study is particularly strong in examining the effects of the early childcare environment on adulthood sociability and its different aspects in that we were able to use a population-based sample with a 32-year follow-up time, with multiple widely used and thoroughly validated sociability indicators, and with several adjusted covariates. Furthermore, we avoided recall bias and some of the problems of common method variance by obtaining information about childhood factors directly from the participants’ parents at the beginning of the study. The extensive study of temporal precedence, potential confounding covariates, and attrition effects alleviates the risk of confounded causality in correlational designs.

Our childcare data dates back to the 1980s when possibilities of a Finnish child to attend municipal daycare were dependent on parental income level. As a result, the majority of 3-year-olds were cared for at home (Säkkinen and Kuoppala, 2011). In the current study, we adjusted for parental SES, but it should nevertheless be noted that children placed in daycare during the 1980s were likely to have a lower parental income compared to children cared for at home. Since 1997, Finnish parents have had a “subjective right to childcare” for children under the age of 3 regardless of family income or parental employment. Nevertheless, the ratio of 3-year-olds placed in outside-home care has changed relatively little in 30 years: in the present study, 43% of 3-year-olds were in outside-home care in 1980 whereas by 2009 the number has increased to 46% (Taguma et al., 2012). For 6-year-olds, we were able to have a more balanced distribution of participants across different forms of childcare than what would be attained in studies of more recent birth cohorts. Nowadays, almost all 6-year-olds attend preschool (Hujala et al., 2012) whereas in our sample the ratio was 36% (in 1983). Therefore, the lack of variation especially among 6-year-olds might preclude replication of the present comparison between early childcare arrangements in contemporary Finland. Data from other countries could be valuable in these respects.

There are, however, limitations in our study that should be considered. The main limitation is that we lacked detailed information about the characteristics of daycare, such as the exact peer-group size or the cumulative hours spent in care outside the home. Moreover, we did not have information about the age of entry into outside-home care or possible changes in care arrangements before the age of 3 or between the ages of 3 to 6. We presented a supplementary analysis of daycare history where we combined daycare at age 3 with care at age 6. However, due to the unbalanced distribution of observations in different daycare history combinations, these results should be cautiously read and would require further studies. We were also unable to separate those 6-year-olds who might have been in preschool from those who were actually cared for in center-based daycares. The blurred lines between these factors may attenuate, but should not change, the general trends we observed, as in 1980s Finland the difference between center-based daycare and preschool was still somewhat inconsequential. Although we controlled for various factors in childhood, child temperament was not directly assessed, and the child’s disruptive behavior can only be regarded as a crude measure for this purpose. More knowledge on childhood temperament would enable investigation in more detail whether those attending childcare differ from those who stay at home until the preschool entry, or, for example, whether sociability in childhood is related to the association between outside-home care and a higher level of adulthood sociability. Although our previous findings in partly the same sample indicate that self- or parent-selection based on the most studied genes would not explain our present results (Oksman et al., 2018), we cannot rule out a selection with respect to other genes. Additionally, from the indicators of sociability used in our study, the low Cronbach’s alpha of Dependence should be noted, as it indicates somewhat poor internal consistency and reliability of the scale in our study sample. This may partly explain why this indicator did not associate with the predictors, but this would need more research.

## Conclusion

First, we found an association between early childcare and higher overall adulthood sociability assessed 32 years later for 3- and 6-year-olds. At age 3, both being in family daycare and being in center-based daycare associated with higher adulthood sociability than being in home care, whereas at age 6, only center-based daycare/preschool associated statistically significantly with differential adulthood sociability. Second, among the indicators of sociability, exposure to outside-home care associated especially with a person’s preference for other people’s company over solitude and his or her tendency to be socially attached to them. Previously, daycare has been shown to predict a wide array of social behavior (e.g., prosocial behavior and compassion), but its role in the development of adulthood sociability is poorly understood. The effect sizes in our study were small, but considering the complexity and a lifespan of humans, it is noteworthy that individual differences in sociability based on the early childcare environment were found in adulthood, after the adjustment of several potential confounding covariates. The findings warrant future studies exploring the mechanisms through which early childcare environments become associated with later sociability.

## Data Availability

These data were used under a data processing agreement in compliance with the GDPR. The raw data for this study is not publicly available, but it may be obtained by contacting the corresponding author. R code for data processing and analyses is available upon a request from the first author (EO). For other requests related to the data please contact Professor Marko Elovainio (marko.elovainio@helsinki.fi) or Adjunct Professor LP-R (laura.pulkki-raback@helsinki.fi).

## Ethics Statement

The study was approved by the local ethics committee, conducted in accordance with the Declaration of Helsinki, and the participants or their parents have provided informed written consent.

## Author Contributions

JV, OR, and LP-R provided the Young Finns data. EO and TR conceived the study and performed the data analysis. EO, TR, and LK-J wrote the manuscript. KG, AS, and MH took part in the writing of the manuscript and interpreting of the findings. All authors discussed the results and reviewed the final manuscript.

## Conflict of Interest Statement

The authors declare that the research was conducted in the absence of any commercial or financial relationships that could be construed as a potential conflict of interest.
