# Supplementary material for: Associations Between Early Childcare Environment and Different Aspects of Adulthood Sociability: The 32-Year Prospective Young Finns Study
Source: Front Psychol. 2019 Sep 10;10:2060. doi: 10.3389/fpsyg.2019.02060 (PMC6746937; doi:10.3389/fpsyg.2019.02060)
Supplement: Supplementary file 1 [file Data_Sheet_1.docx]

Supplementary Material

# Supplementary analysis of childcare history as a predictor for the overall adulthood sociability and different aspects of sociability.

**Table S1.** Multilevel regression analyses of early childcare history (i.e., care environment at age 3 and 6) predicting standardized overall adulthood sociability (*N* = 9280).

| Early childcare history | |  |  |  |  |
| --- | --- | --- | --- | --- | --- |
| At age 3 | At age 6 | n (%) | β | 95% CI | *p*-value |
| Home care | Home care | 3500 (38.7) | (Ref) |  |  |
| Home care | Family care | 360 (3.9) | .11 | -0.16 to 0.36 | .493 |
| Home care | Center-based care | 840 (9.1) | .12 | -0.04 to 0.32 | .384 |
| Family care | Home care | 500 (5.4) | .18 | -0.12 to 0.34 | .174 |
| Family care | Family care | 1240 (13.4) | .17 | 0.04 to 0.36 | .010 |
| Family care | Center-based care | 1200 (12.9) | **.29**** | 0.08 to 0.41 | **.002** |
| Center-based care | Home care | 160 (1.7) | .04 | -0.08 to 0.68 | .877 |
| Center-based care | Family care | 220 (2.4) | .18 | -0.17 to 0.47 | .450 |
| Center-based care | Center-based care | 1260 (13.6) | **.28**** | 0.07 to 0.39 | **.003** |
| ***Note.*** The statistically significant values are bolded, ** = *p* < .01. The *p*-value indicates the difference from the day care history when the child have been cared in home care both at age 3 and 6 which was set as a reference group. Model was adjusted for gender, disruptive behavior in childhood, parental socio-economic status, parent-child relationship quality, maternal age, and the number of children in the family. All the values presented are based on estimates employing missing data modeling. | | | | | |

**Table S2.** Multilevel regression analyses of early childcare history (i.e., care environment at age 3 and 6) predicting different aspect of adulthood sociability (*N_y[i]_* = 1 856 for each aspect).

| Early childcare history | |  | Aspects of adulthood sociability | | | | |
| --- | --- | --- | --- | --- | --- | --- | --- |
| At age 3 | At age 6 |  | β | 95% CI | | | *p*-value |
|  |  |  | TCI RD1 Sentimentality | | | | |
| Home care | Home care |  | (Ref) |  | | |  |
| Home care | Family care |  | 0.10 | -0.33 to 0.26 | | | .600 |
| Home care | Center-based care |  | 0.16 | -0.05 to 0.35 | | | .260 |
| Family care | Home care |  | 0.12 | -0.13 to 0.38 | | | .455 |
| Family care | Family care |  | 0.09 | -0.05 to 0.32 | | | .430 |
| Family care | Center-based care |  | 0.13 | -0.10 to 0.27 | | | .288 |
| Center-based care | Home care |  | 0.16 | -0.03 to 0.82 | | | .534 |
| Center-based care | Family care |  | 0.10 | -0.18 to 0.55 | | | .689 |
| Center-based care | Center-based care |  | **0.20*** | -0.04 to 0.32 | | | **.050** |
|  |  |  | TCI RD3 Social attachment | | | | |
| Home care | Home care |  | (Ref) |  | | |  |
| Home care | Family care |  | 0.23 | | -0.30 to 0.56 | .393 | |
| Home care | Center-based care |  | 0.16 | | -0.13 to 0.46 | .435 | |
| Family care | Home care |  | 0.33 | | -0.14 to 0.60 | .116 | |
| Family care | Family care |  | 0.32 | | 0.02 to 0.55 | .068 | |
| Family care | Center-based care |  | **0.49**** | | 0.15 to 0.68 | **.003** | |
| Center-based care | Home care |  | 0.43 | | 0.39 to 1.63 | .270 | |
| Center-based care | Family care |  | 0.41 | | -0.24 to 0.82 | .287 | |
| Center-based care | Center-based care |  | **0.42*** | | 0.11 to 0.64 | **.012** | |
|  |  |  | TCI RD4 Dependence | | | | |
| Home care | Home care |  |  | |  |  | |
| Home care | Family care |  | 0.14 | | -0.10 to 0.48 | .454 | |
| Home care | Center-based care |  | 0.09 | | -0.11 to 0.29 | .545 | |
| Family care | Home care |  | 0.03 | | -0.23 to 0.28 | .855 | |
| Family care | Family care |  | 0.03 | | -0.06 to 0.31 | .822 | |
| Family care | Center-based care |  | 0.13 | | -0.02 to 0.34 | .222 | |
| Center-based care | Home care |  | -0.18 | | -0.41 to 0.44 | .491 | |
| Center-based care | Family care |  | 0.12 | | -0.43 to 0.30 | .672 | |
| Center-based care | Center-based care |  | 0.11 | | -0.10 to 0.25 | .342 | |
|  |  |  | EAS Sociability | | | | |
| Home care | Home care |  | (Ref) |  | | |  |
| Home care | Family care |  | 0.03 | -0.32 to 0.56 | | | .912 |
| Home care | Center-based care |  | 0.10 | -0.08 to 0.52 | | | .632 |
| Family care | Home care |  | 0.36 | -0.15 to 0.61 | | | .115 |
| Family care | Family care |  | 0.29 | 0.06 to 0.61 | | | .090 |
| Family care | Center-based care |  | **0.39*** | 0.08 to 0.63 | | | **.011** |
| Center-based care | Home care |  | -0.17 | -0.64 to 0.63 | | | .670 |
| Center-based care | Family care |  | 0.11 | -0.42 to 0.66 | | | .780 |
| Center-based care | Center-based care |  | **0.42**** | 0.12 to 0.66 | | | **.008** |
|  |  |  | NEO Extraversion | | | | |
| Home care | Home care |  | (Ref) |  | | |  |
| Home care | Family care |  | 0.04 | -0.36 to 0.43 | | | .860 |
| Home care | Center-based care |  | 0.03 | -0.25 to 0.28 | | | .886 |
| Family care | Home care |  | 0.07 | -0.42 to 0.25 | | | .738 |
| Family care | Family care |  | 0.14 | -0.11 to 0.37 | | | .355 |
| Family care | Center-based care |  | 0.28 | -0.03 to 0.45 | | | .058 |
| Center-based care | Home care |  | -0.05 | -0.38 to 0.72 | | | .872 |
| Center-based care | Family care |  | 0.17 | -0.22 to 0.73 | | | .628 |
| Center-based care | Center-based care |  | 0.25 | -0.04 to 0.43 | | | .071 |
| ***Note.*** *N* = 1856. The statistically significant values are bolded, * = *p* <.05, ** = *p* < .01. The *p*-value indicates the difference from the day care history when the child have been cared in home care both at age 3 and 6 which was set as a reference group. Model was adjusted for gender, disruptive behavior in childhood, parental socio-economic status, parent-child relationship quality, maternal age, and the number of children in the family. All the values presented are based on estimates employing missing data modeling. | | | | | | | |
